# Fractionation of Fab glycosylated immunoglobulin G with concanavalin A chromatography unveils new structural properties of the molecule

**DOI:** 10.18632/oncotarget.9085

**Published:** 2016-04-28

**Authors:** Tao Huang, Xueling Chen, Huan Gu, Conghui Zhao, Xingmu Liu, Meiling Yan, Xiaodong Deng, Zaiping Zhang, Jiang Gu

**Affiliations:** ^1^ Department of Pathology and Provincial Key Laboratory of Infectious Diseases and Immunopathology, Collaborative and Creative Center, Molecular Diagnosis and Personalized Medicine, Shantou University Medical College, Shantou, Guangdong, 515041, China; ^2^ Department of Oral Pathology, Beijing Stomatological Hospital, Capital Medical University, Beijing, 100050, China; ^3^ Department of Pathology, Beijing University Health Science Center, Beijing, 100083, China; ^4^ Department of General Surgery, Second Affiliated Hospital, Shantou University Medical College, Shantou, Guangdong, 515041, China

**Keywords:** IgG, glycosylation, ConA, asymmetry, symmetry

## Abstract

Concanavalin A (ConA) chromatography has been extensively used to separate asymmetric Immunoglobulin G (IgG), which possesses oligosaccharide attached to one of the two F(ab')_2_ arms, from symmetric IgG with no glycan attached to Fab fragments. In this study, applying affinity chromatography, silver stain, Western blot and lectin stain techniques, N- linked oligosaccharide attached to Fab fragment was demonstrated to be exposed on the surface of the protein and be accessible by ConA. In contrast, N- linked oligosaccharide attached to asparagine (Asn) 297 of IgG Fc was located in the inside of the natural protein and was inaccessible by ConA. In addition to asymmetric IgG, there are also detectable level of IgG with both F(ab')_2_ arms glycosylated that has not been reported previously. The discoveries of new basic molecular structure of IgG would have implications in understanding the function and properties of this important immune molecule with clinical applications.

## INTRODUCTION

**Figure 1:**
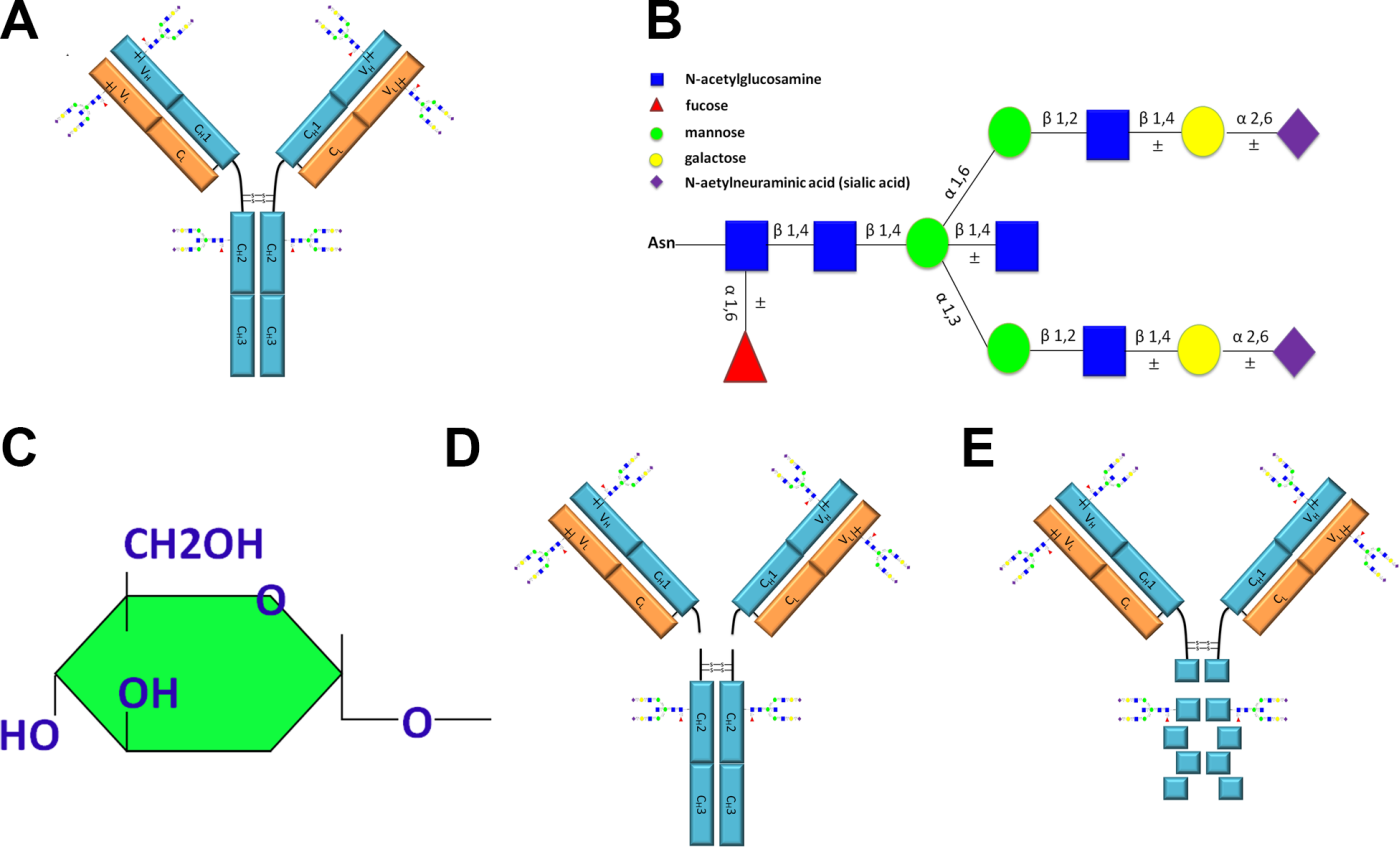
Schematic representation of IgG molecular structure including the glycoforms attached to IgG, the sugar residue that is recognizable by ConA and fragments of IgG obtained with enzyme digestion (**A**) The molecular structure of IgG and potential glycosylation sites. The IgG Fc C_H_2 domains are conserved and are glycosylated through attachment of oligosaccharides at Asn 297. In addition to this conserved glycosylation site, extra oligosaccharides may be attached to the variable regions of the light chain, the heavy chain or both. (**B**) Schematic representation of the glycoforms attached to IgG. (**C**) The structure of sugar residue that can be specifically recognized by ConA. (**D**) Fab and Fc fragments derived from IgG with papain digestion. (**E**) F(ab')_2_ and degraded Fc fragments obtained from IgG with pepsin digestion. ±, represents possible glycosylation sites or additional sugar residues.

IgG has long been known as a glycoprotein with two highly conserved glycosylation sites located at Asn 297 in the C_H_2 domains of the Fc region. Further oligosaccharides may be found attached to the variable regions of IgG molecules [1]. The molecular structure of IgG is shown schematically in Figure 1A. The Fc glycosylation has been extensively studied to illustrate glycans' structure, influence on antibodies' function and glycoform changes associated with diseases. The common characteristic of IgG Fc oligosaccharides is of the complex type with a constant heptasaccharide core. Additional sugar residues such as core fucose (Fuc), bisecting N-acetylglucosamine (GlcNAc), galactose(s) (Gal), and N-aetylneuraminic acid(s) (NeuAc) may attached to the “core” [2] (Figure 1B).

In addition to Asn 297 of Fc fragment, 15– 20% of natural human IgG molecules bear N-linked oligosaccharides within the Fab fragments [3–5]. There are no glycosylation sites within the constant domains of either the light chain (κ- or λ- chain) or the C_H_1 domain of heavy chains. Therefore, the oligosaccharides attach to the variable regions of the light chain (V_κ_ or V_λ_), heavy chain (V_H_) or probably both. Attachment of the carbohydrates to the variable regions of light chains have been found in IgG from patients with multiple myeloma and an anti-ovomucoid monoclonal antibody (MAb-OM21) [6, 7]. The glycans attach to the heavy chain variable regions of the antibody specific for α(1→6) dextran (TKC3.2.2) and its effects on antigen-binding affinity have been evaluated [8, 9]. The glycoforms attached to the Fab regions are of complex type with higher levels of galactorsylation and sialylation than those of Fc glycans [10, 11].

Lectins are useful tools for fractionation and structural analysis of oligosaccharides and glycoproteins. ConA has long been used for separation of symmetric and asymmetric IgG molecules [12]. The symmetric IgG was thought to be Fab non-glycosylation fraction and the asymmetric IgG was the fraction with additional carbohydrate residue present in only one heavy chain variable region of two F(ab')_2_ arms. ConA is a tetrameric metalloprotein and specifically binds molecules containing the structure of sugar residue shown in Figure 1C [13]. Consequently, sugar residues which occur at non-reducing termini of sugar chains such as glucose α1→, Manα→ and GlcNAcα1→ residues and sugar residues which occur within sugar chains such as →2glucose α1→ and →2Manα1→ residues can bind to this lectin. The →β2 Man α1→ residue conserved located in heptasaccharide core of the complex type oligosaccharides, therefore, by theory, all IgG molecules ought to react to ConA because of N-glycans attached to Asn 297 of IgG Fc. In reality, however, only 12% of natural human IgG bound to ConA column [12] with reasons unknown. This study resolved this paradox and unveiled that ConA was bound to N- linked oligosaccharides attached to Fab regions that were exposed on the surface of IgG and were accessible by ConA. In contrast, N- linked oligosaccharides atattached to Asn 297 of IgG Fc were located in the inside of the natural protein and were inaccessible by ConA. In addition, we further demonstrated that not all Fab glycosylated IgGs were asymmetric. Fab symmetrically glycosylated IgG, of which both arms of F(ab')_2_fraction were glycosylated, was successfully fractioned with ConA chromatography. This study does not only establish an effective method to fractionate Fab glycosylated IgG into three forms, i.e. Fab non-glycosylation IgG, Fab asymmetrically glycosylated IgG and Fab symmetrically glycosylated IgG with ConA affinity chromatography, but also unveil new facts about the basic structure and function of IgG molecule with theoretical and practical implications.

## RESULTS

### The molecular characteristic of ConA+ IgG

**Figure 2:**
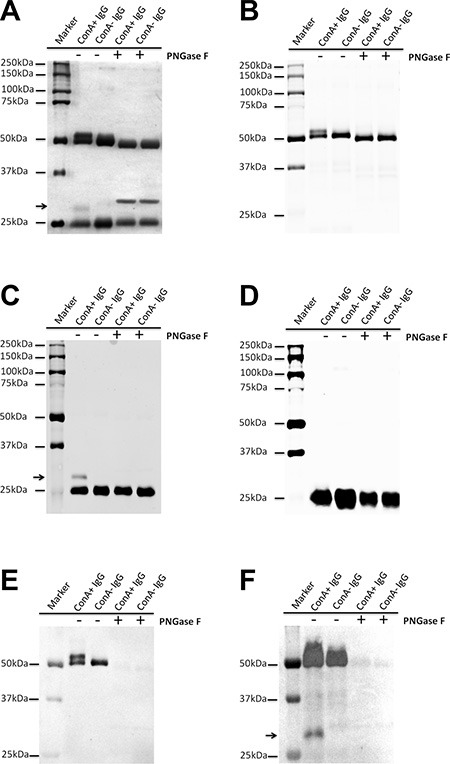
Extra N-glycans attach to part heavy chain or kappa light chain of ConA+ IgG (**A**) Silver stain shows that there are higher molecular weight bands above the 50 kDa heavy chain and the 25 kDa light chain (arrow), which return to the normal sizes following treatment with deglycosylation enzyme PNGase F. (**B–D**) Western blot established that the higher molecular weight band above the 50 kDa heavy chain was Ig γ heavy chain (B), the higher molecular weight band above the 25 kDa (arrow) was kappa light chain (C), but not lambda light chain (D). (**E** and **F**) Lectin stain shows that both heavy chain of ConA+ and ConA- IgG under denaturation and reduction conditions could react with ConA. Neither kappa nor lambda light chain could react with ConA except the higher molecular weight band above the 25 kDa (arrow). The difference between E and F is in sample quantity, E (0.5 μg/well) and F (1.5 ug/well). The sample quantity of A-D was 1 ug/well.

Human IgG was purified from serum with Protein G column and then fractionated with ConA Sepharose 4B. The difference in molecular weight of ConA+ and ConA- IgG was examined with electrophoresis and silver stain (Figure 2A). Additional higher molecular weight bands existed above the 50 kDa heavy chain band and the 25 kDa light chain band in ConA+ IgG fraction and these two bands returned to the normal sizes following treatment with deglycosylation enzyme PNGase F. This indicates that the two higher molecular weight bands might be heavy chain and light chain with extra N-glycans attaching to them. In order to determine the identity of these higher molecular weight bands, Western blot was performed with anti-Ig γ Fc region, anti-Ig κ light chain and anti-Ig λ light chain antibodies. As shown in Figure 2B, the higher molecular weight monomer above the 50 kDa heavy chain indeed was IgG heavy chain that can be identified with anti-Ig γ Fc region antibody. Figure 2C, Figure 2D and Table 1 show that the higher molecular weight monomer above the 25 kDa light chain was Ig κ light chain. There are carbohydrates attached to the λ light chain of human γG1 myeloma proteins from the urine of patients with multiple myeloma has been reported [14]. However, no detectable level of N-glycans attached to λ light chain of natural human IgG was found in this study.

**Table 1:** The band of arrow points to (correspond to Figure 2) was determined with MALDI TOF MS analysis

In-gel digestion	Protein hits	Mascot score
The band ofarrow points to	Ig kappa chain C region	56
	Ig kappa chain V-IV region	41

### Oligosaccharides attached to Asn 297 are located in the inside of the molecule in its natural state

**Figure 3:**
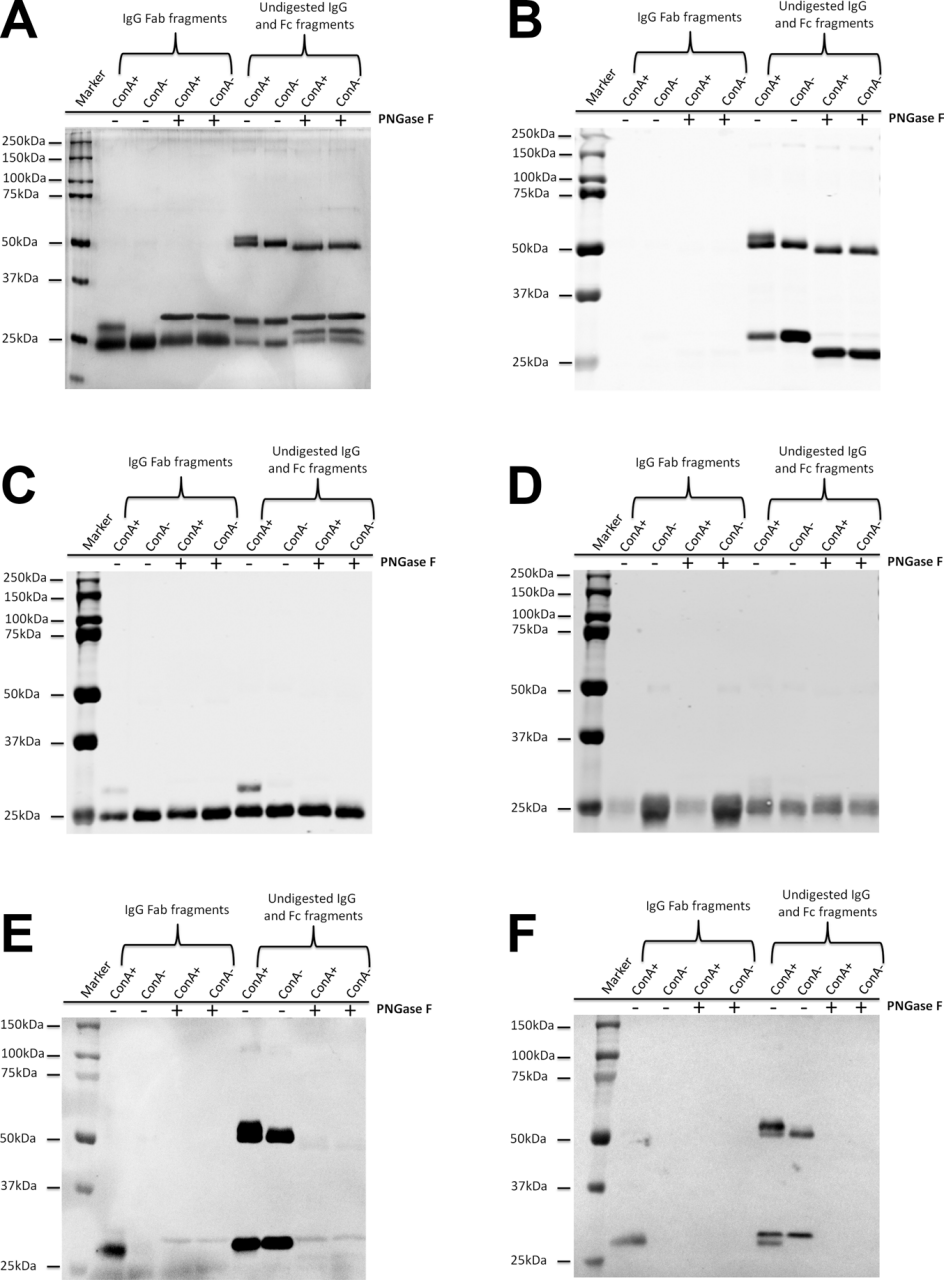
Extra N-glycans exist in Fab fragments but not in Fc fragments of ConA+ IgG (**A**) Silver stain shows extra higher molecular weight band above the denatured and reduced Fab fragments but not above the denatured and reduced Fc fragments of ConA+ IgG. The higher molecular weight band returned to the normal size following treatment with deglycosylation enzyme PNGase F. (**B–D**) Western blot established that the higher molecular weight band above the denatured and reduced Fab fragments was a mixture of IgG heavy chain Fab fraction but not Fc fraction (B) and kappa light chain (C) but not lambda light chain (D). (**E**) ConA stain shows that both the higher molecular weight Fab fragments and the Fc fragments of ConA+ and ConA- IgG under denaturation and reduction conditions could react with ConA. (**F**) SNA stain shows that sialic acids are α-2,6- linked to oligosaccharides in both Fab fragments and Fc fragments.

**Figure 4:**
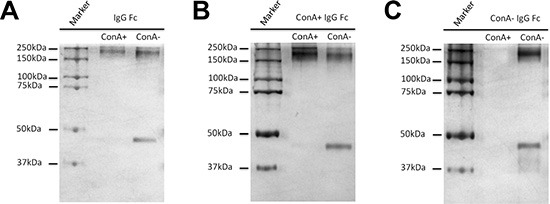
Oligosaccharides attached to Asn 297 of IgG Fc fragments are located in the inside of the natural protein of IgG and are inaccessible by ConA Fc fragments (contain undigested IgG) obtained from un-fractionated IgG (**A**), ConA+ IgG (**B**) and ConA- IgG (**C**) were loaded onto ConA column. The bound and unbound fractions were collected and then examined with silver stain. The Fc fragments under non-reducing condition, regardless if they were obtained from un-fractionated IgG (A), ConA+ IgG (B) or ConA- IgG (C), could not react with ConA indicating that oligosaccharides attached to Asn 297 were in the inside of the natural protein structure of IgG and were inaccessible by ConA.

**Figure 5:**
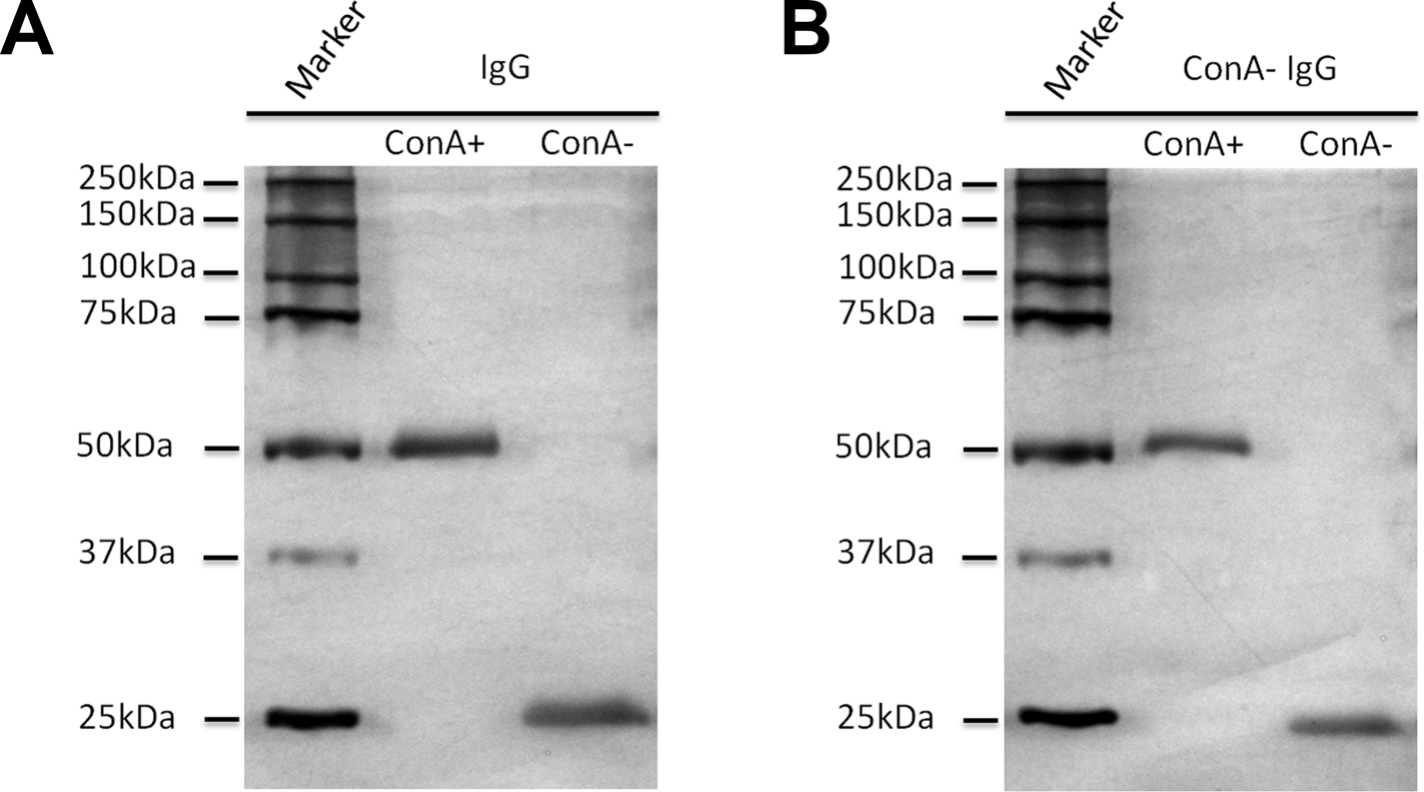
All Igγ heavy chains bind to ConA column following denaturation and reduction with SDS and β-mercaptoethanol IgG (**A**) and ConA- IgG (**B**) were loaded onto ConA chromatography after denaturation and reduction, and then examined with silver stain. The heavy chain of IgG (A) including ConA- IgG (B) could bind with ConA due to glycans attached to Asn 297 that were exposed after denaturation and reduction. The 25 kDa band of light chain could not bind to ConA column as no oligosaccharide was attached to it.

The location of oligosaccharides of IgG was examined with affinity chromatography, silver stain, Western blot and lectin stain techniques. As shown in Figure 2E–2F, after denaturation and reduction with SDS and β-mercaptoethanol both ConA- and ConA+ IgG reacted with ConA indicating that the oligosaccharides attached to Asn 297 of Fc should be in the inside of the 3D protein structure and shielded from ConA. The reaction of IgG Fc fragments with ConA was also observed with lectin stain after papain digestion (Figure 3E). Under natural state, there was no reaction between ConA and Fc no matter if the Fc was derived from IgG, ConA+ IgG or ConA- IgG (Figure 4A–4C). On the contrary, all IgG heavy chains bound to ConA after denaturation and reduction (Figure 5A–5B).

### Oligosaccharides attached to Fab fragments are responsible for the binding of IgG to ConA

In electrophoresis, ConA+ IgG Fab fraction generates a band with higher molecular weight that would fall back to the normal molecular weight following treatment with deglycosylation enzyme PNGase F. In contrast, no molecular weight difference was detected between ConA+ and ConA- IgG Fc fragments (Figure 3A). Most of the higher molecular weight band in ConA+ IgG Fab fraction was Fd fragment, and a relatively small percentage was κ light chain (Figure 3B–3D).

As shown in Figure 3E, no ConA reactive Fab fragment was detected in ConA- IgG Fab fraction suggesting that all Fab glycosylated IgG molecules were collected into ConA+ IgG fraction. This further suggests that oligosaccharides attached to Fab fragments are likely exposed on the surface of the protein and can be bound by ConA. In addition, sialylation of N-linked oligosaccharides attached to IgG Fab and IgG Fc fragments both could be detected with SNA stain (Figure 3F).

### Effective fractionation of IgG with different degrees of Fab glycosylation

**Figure 6:**
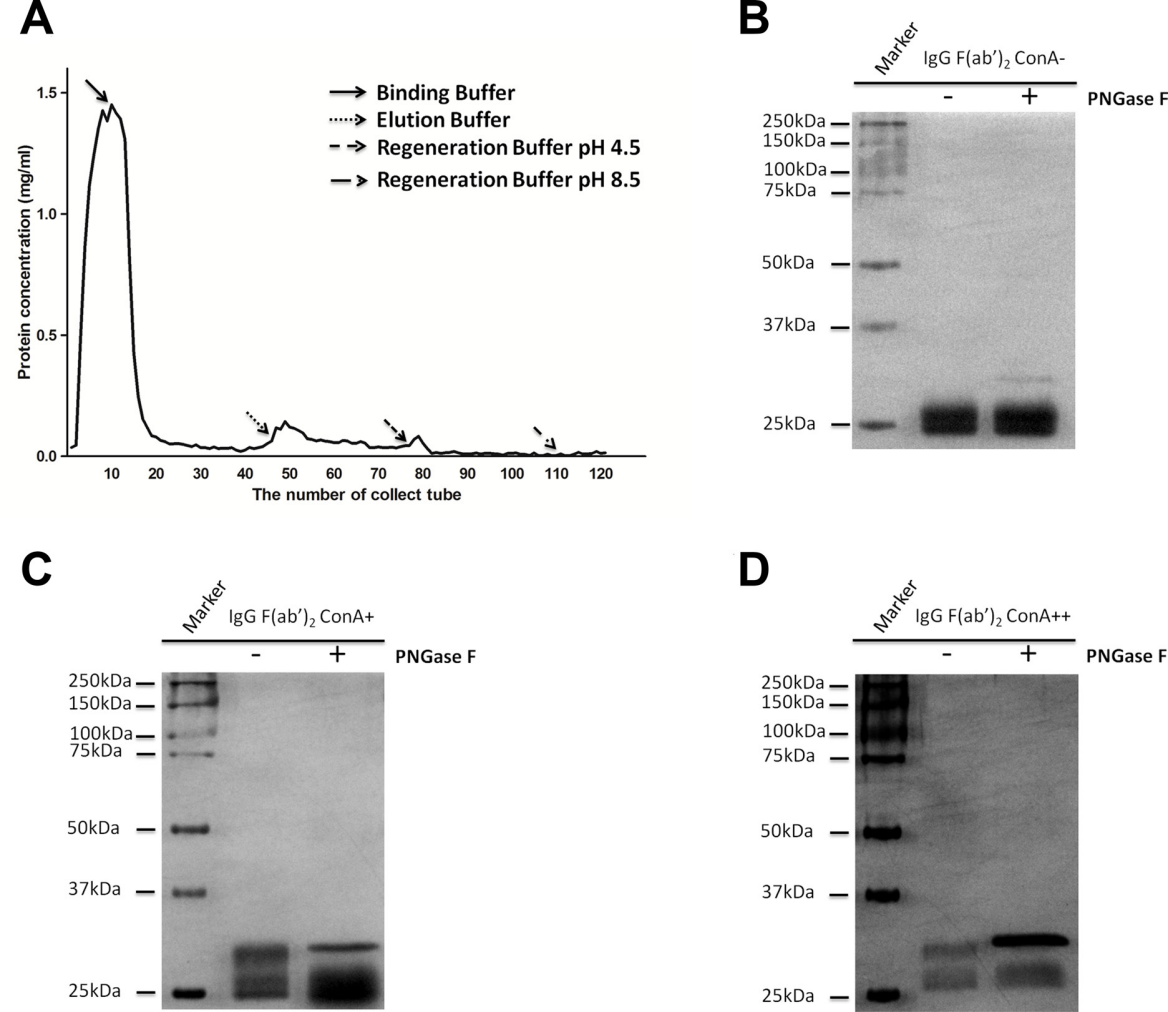
IgG was fractionated into three fractions: Fab non-glycosylation IgG (ConA- IgG), Fab asymmetrically glycosylated IgG (ConA+ IgG) and Fab symmetrically glycosylated IgG (ConA++ IgG) with ConA affinity chromatography (**A**) Three fractions of IgG were isolated by eluting ConA column with different solutions including ConA- IgG eluted with Binding Buffer, ConA+ IgG eluted with Elution Buffer and ConA++ IgG eluted with Regeneration Buffer pH 4.5. (**B–D**) F(ab')_2_ fragment of IgG was loaded onto ConA column, and its three fractions were examined with silver stain with or without PNGase F digestion. (B) No molecular weight decline was observed for ConA- F(ab')_2_ fraction after PNGase F digestion and this suggests that no N-glycans were attached to F(ab')_2_ fraction. (C) When only half of Fd' fragments of ConA+ F(ab')_2_ fraction were found to decline in molecular weight after PNGase F digestion, it was suggested that N-glycans were attached to F(ab')_2_ fraction in only one arm asymmetrically. (D) When all Fd' fragments of ConA++ F(ab')_2_ fraction declined in molecular weight after PNGase F digestion suggested that N-glycans were attached to both arms of F(ab')_2_ fraction symmetrically.

An IgG molecule is composed of two identical light chains and two identical heavy chains, and form a structure with twofold symmetry [15]. When one of two F(ab')_2_ arms is glycosylated it is named asymmetry IgG [12]. The explanation for this phenomenon is that the competitive reactions of posttranslational modification and protein folding may result in the glycosylation of only a portion of the variable region [16]. In this study, we attempted to ascertain if there exist a form of Fab symmetrically glycosylated IgG of which both F(ab')_2_ arms are glycosylated. To clarify this supposition, we loaded 10 mg IgG onto ConA column, eluted with different solutions, collected the eluent at 0.5 mL per fraction and then determined the concentrations with absorbance at A_280_. As shown in Figure 6A, three main fractions of IgG were isolated. The characteristics of these three fractions are shown in Figure 6B–6D. No molecular weight decline of ConA- IgG F(ab')_2_ fraction after PNGase F digestion indicates non-glycosylation of the F(ab')_2_ fragment (Figure 6B). A decline of one half of Fd' fragments of ConA+ IgG F(ab')_2_ fraction in molecular weight after PNGase F digestion suggests the existence of Fab asymmetrically glycosylated IgG (Figure 6C). A decline of all Fd' fragments of ConA++ IgG F(ab')_2_ fraction in molecular weight after PNGase F digestion implies the existence of Fab symmetrically glycosylated IgG (Figure 6D).

## DISCUSSION

Immobilized lectin columns have been used extensively for the fractionation and structural analysis of oligosaccharides and glycopeptides [13, 17]. For example, SNA specifically recognizes the Neu5Ac(α2-6)Gal/GalNAc sequence [18]. MAA has affinity to oligosaccharides containing terminal sialic acid (α2-3) linked to Gal/GalNAc [19]. The carbohydrate binding property of GNA is terminally linked to mannose specifically [20]. DSA recognizes Gal-β(1-4)-GlcNAc without terminal sialic acids [21]. It was demonstrated that unmodified hydroxyl groups at the C-3, C-4, and C-6 positions of a glycopyranosyl residue are essential for the interaction of ConA [22]. IgG molecules are glycoproteins containing at least N-linked carbohydrates in their Fc fragments [23]. The structure of oligosaccharides attached to Asn 297 of IgG Fc fragments is largely conserved, and in theory, the →β2Manα1→residue located in heptasaccharide core of the biantennary complex type oligosaccharides should be recognized by ConA (Figure 1B). In reality, however, only 12% of natural human IgG bound to ConA column [12]. The present study resolved this paradox, we demonstrated that the Fc-linked oligosaccharides are located in the inside of the natural protein and are inaccessible by ConA. In contrast, oligosaccharides attached to Fab fragments are exposed on the surface of the protein and contribute to the binding with ConA column.

It has been known for years, based on investigation with crystallography, that oligosaccharides attached to Asn 297 of IgG Fc fragments are largely located internally in the secondary/tertiary structure of the molecule [24]. However, this internal location does not completely prevent it from binding to external molecules. For example, the glycans on the Fc region of IgG could bind to SNA if there are two or more sialic acids [25]. It is possible that different molecule conformations of mono- and disialylations are for the reasons for this differences, with one sialic acid hidden therefore inaccessible, and the two sialic acid residues exposed therefore accessible by SNA. The accessibility of ConA to Fc glycans, however, appears to be different. We demonstrated that the internal location of β2 Man α1→ residue in native Fc region was inaccessible by ConA regardless of the glycoforms. The accessibility of other lectins to Fc glycans remain a subject of farther investigation.

A kinetic explanation was proposed for the occurrence of Fab asymmetric glycosylation. Protein folding and posttranslational modification are the competitive reactions may result in only one arm of of F(ab')_2_ fraction being glycosylated [16]. Likewise, there might be Fab symmetric glycosylation IgG with both F(ab')_2_ arms being glycosylated. In this study, we demonstrated the existence of such IgG portion which was eluted with Regeneration Buffer pH 4.5. This experiment data not only verify the kinetic explanation of Fab glycosylation but also offers a technique to fractionate Fab asymmetrically glycosylated IgG and Fab symmetrically glycosylated IgG effectively.

The structure of Fc glycoforms, their influence on IgG function and their association to diseases have been studied extensively. Higher levels of sialylated Fc glycans were associated with reduced ADCC activity because of lower affinity binding to FcγRIIIa and/or lower affinity binding to cell surface antigens [26]. Sialylation of the N-linked glycan of the IgG Fc fragment completely determined the anti-inflammatory activity of IgG [27, 28]. The ADCC was enhanced by removing Fuc (fucose) from complex type oligosaccharides [29–32]. The GlcNAc of oligosaccharides in the Fc region binding to the collagenous lectin mannose-binding protein (MBP) contribute to a new mode of interaction of IgG with complement [33]. The changes in glycosylation patterns have also been found to be associated with diseases and aging [34–39]. Nevertheless, it should be noted that recent researches indicated that the sialylation IgG obtained by SNA fractionation with anti-inflammatory activity was essentially mediated by Fab not by Fc sialylation [25, 40]. Combining with our current findings, we have reason to believe that some of previous conclusions about the function of Fc glycans, especially those established on the fractionation of IgG with lectin column, should be re-evaluated due to the intramolecular location of Fc glycans. Further investigations would be necessary to determine whether certain Fc glycan functions reported previously are in fact mediated by Fab glycosylation.

Fewer studies have been done to study IgG Fab glycans comparing with Fc glycans. The Fab glycosylation is presumed to be present on the variable region of heavy or light chain due to neither the light chain nor the heavy chain C_H_1 region contains a carbohydrate binding sequence (Asn-X-Ser/Thr; where X can be any amino acid except proline) [1, 41]. In a series of early studies, carbohydrate addition sites were found located in the variable region of myeloma, Bence-Jones and amyloid fibril proteins [6, 14, 42–44]. While the function of IgG Fab glycosylation has not been fully evaluated, its influence on antigen binding has been well established with monoclonal antibodies [7–9, 45]. Only a few associations of glycoforms of IgG Fab fragments with diseases (e.g. ANCA-associated systemic vasculitis) and with pregnancy have been reported [4, 46]. Recently, a series of high-throughput, sensitive methods have been established to effectively analyze IgG Fab glycans with mass spectrometry [46] or capillary electrophoresis [47, 48]. These methods have become powerful tools for IgG Fab glycosylation analysis, however, they cannot successfully fractionate whole Fab glycosylated IgG molecules from IgG mixtures. The present study provides an effective method to fractionate Fab glycosylated IgG, that could accurately distinguish Fab asymmetrically glycosylated IgG and Fab symmetrically glycosylated IgG with significant scientific and clinical implications.

## MATERIALS AND METHODS

### Ethics statement

The Ethical Committee of Shantou University Medical College has approved all experiments. All donors have provided written informed consent.

### Isolation of IgG from human serum

Human serum was obtained through centrifuging healthy adult donors' peripheral blood at 500 × g for 5minutes at 4°C. Total IgG was purified from serum using affinity chromatography with Recombinant Protein G Agarose according to the manufacturer's instructions (Invitrogen, California, USA).

### ConA affinity chromatography

Human IgG was fractionated with lectin affinity chromatography using ConA Sepharose 4B following the manufactuer's description (GE Healthcare, Connecticut, USA). In brief, IgG was diluted with Binding Buffer (20 mM Tris-Hcl pH 7.4 containing 0.5 M NaCl, 1 mM CaCl_2_, 1 mM MgCl_2,_and 1 mM MnCl_2_) and loaded onto 2 mL of Sepharose 4B-linked ConA column. The column was further washed with Binding Buffer to remove the unbound IgG, and all these unbound fractions were mixed in order to obtain the unbound IgG named ConA- IgG. The fraction bound to the ConA column was orderly eluted with Elution Buffer (25 mM Tris-Hcl pH 7.4 containing 0.2 M NaCl and 0.15 M α-D-methylmannoside) and Regeneration Buffer pH 4.5. The fraction isolated by elution with Elution Buffer was named as ConA+ IgG, and the fraction isolated by elution with Regeneration Buffer pH 4.5 was named as ConA++ IgG. The affinity chromatography of denatured IgG, Fab fragment, Fc fragment and F(ab')_2_ fragment were performed identically to IgG. The concentration of all fractions were performed with Amicon Ultra-4 Cnetrifugal Filter Units, 10kDa (Millipore, Massachusetts, USA) and the concentrations were determined with absorbance at A_280_.

### Production of IgG fragments

Fab and Fc fragments were produced from IgG using Pierce Fab Preparation kit and F(ab')_2_ fragment were prepared with Pierce F(ab')_2_ Preparation kit following the manufacturer's instructions (Pierce Biotechnology, Illinois, USA).

### Deglycosylation enzymatic treatment

The deglycosylation enzymatic treatment of IgG and its Fab, Fc or F(ab')_2_ fragments was performed according to manufacturer's instructions of PNGase F (NEB, Massachusetts, USA).

### SDS PAGE

The IgG and its Fab, Fc or F(ab')_2_ fragments with or without PNGase F treatment were separated with SDS PAGE using 10% polyacrylamide BisTris gels under non-reducing or reducing conditions. The gels were then stained with silver or transferred to nitrocellulose membranes (Whatman, Dassel, Germany) and then examined with Western blot or lectin stain as described below.

### Silver stain

After SDS PAGE, the gels were fixed with fixation fluid (10% acetic acid and 40% ethanol) for 30 minutes to overnight. The gels were incubated with 30% ethanol containing 0.5 M sodium acetate anhydrous and 0.02 M sodium thiosulfate for 30 minutes. Staining was performed with staining buffer (0.01 M silver nitrate combining with 0.05% formaldehyde) for 40 minutes after washing three times in deionized water for 10 minutes each. The gels were developed with a solution containing 0.24 M sodium carbonate anhydrous and 0.05% formaldehyde. The developing process was terminated with 5% acetic acid and the gels were preserved in deionized water.

### Western blot

Protein aliquots were electrophoresed on SDS PAGE and then transferred to nitrocellulose filter membrane (Whatman, Dassel, Germany). The blots were blocked with 5% skim milk in TBST (10 mM Tris Base, 150 mM NaCl, 0.1% Tween-20, pH 8.0) for 1 hour at room temperature, and incubated overnight with primary antibodies. Rabbit anti-human IgG γ Fc region antibody (Dako, Copenhagen, Denmark), mouse anti-human Ig κ chain antibody (ZSGB-BIO, Beijing, China) and mouse anti-human Ig λ chain antibody (ZSGB-BIO, Beijing, China) at a dilution of 1:1,000 were used as primary antibodies. Goat anti-rabbit IgG-680 (1:10,000; LI-COR, Nebraska, USA) and goat anti-mouse IgG-680 (1:10,000; LI-COR, Nebraska, USA) were used as secondary antibodies. The blots were examined with a Odyssey imaging system (LI-COR, Nebraska, USA).

### Lectin stain

For ConA stain, the membrane was blocked in Carbo-Free Blocking Solution (Vector labs, California, USA) for 1 hour and incubated with 2 μg/ml biotin labeled ConA (Vector labs, California, USA) for 1 hour. After washing with TPBS (0.01 M PBS, pH 7.4, 0.05% Tween-20), incubated the membrane with VECTASTAIN ABC-AP kit (Vector labs, California, USA) for 30 minutes according to the kit instructions. BCIP/NBT substrate kit (Vector labs, California, USA) was used for development. All procedures were performed at room temperature.

For SNA (Sambucus nigra agglutinin) stain, experiment was performed using Dig Glycan Differentiation Kit following the manufacturer's instructions (Roche, Basel, Switzerland).

### Protein identification with mass spectrometry

After silver stain of the SDS-PAGE gels, the object bands were cut out. Protein samples were identified with MALDI TOF MS (Matrix-Assisted Laser Desorption/Ionization Time of Flight Mass Spectrometry) analysis.

## Abbreviations

Asn 297: asparagine 297
ConA: Concanavalin A
Gal: galactose
GlcNAc: N-acetylglucosamine
IgG: Immunoglobulin G
Man: mannose
